# Characterization of Non-Anthocyanic Flavonoids in Some Hybrid Red Grape Extracts Potentially Interesting for Industrial Uses

**DOI:** 10.3390/molecules201018095

**Published:** 2015-10-02

**Authors:** Mirko De Rosso, Annarita Panighel, Antonio Dalla Vedova, Massimo Gardiman, Riccardo Flamini

**Affiliations:** Consiglio per la Ricerca in Agricoltura e l’Analisi dell’Economia Agraria—Centro di Ricerca per la Viticoltura (CREA-VIT), Viale XXVIII Aprile 26, Conegliano (TV) 31015, Italy; E-Mails: mirko.derosso@gmail (M.D.R.); annaritapanighel@libero.it (A.P.); antonio.dallavedova@entecra.it (A.D.V.); massimo.gardiman@entecra.it (M.G.)

**Keywords:** antioxidants, hybrid grapes, polyphenols, high-resolution mass spectrometry

## Abstract

Previous studies showed that hybrid grapes often have qualitatively and quantitatively higher polyphenolic contents than the common *V. vinifera* grape varieties. In general, these compounds are studied for grape chemotaxonomy and for nutraceutical purposes due to their relevant antioxidant activity. Non-anthocyanic flavonoid composition of five red hybrid grape varieties produced by crossing of *V. vinifera*, *V*. *aestivalis*, *V. cinerea*, *V. berlandieri*, *V. labrusca*, *V. lincecumii*, and *V. rupestris* were studied by liquid chromatography/high-resolution mass spectrometry. Thirty-one compounds were identified, including methylnaringenin, a tetrahydroxy-dimethoxyflavanone-hexoside, two flavonols (quercetin and a pentahydroxyflavone isomer), 20 glycoside flavonols (four quercetin, two myricetin, two kaempferol, three isorhamnetin, one laricitrin, two syringetin, one kaempferide and two dihydroflavonol derivatives; myricetin-glucoside-glucuronide; myricetin-diglucoside; syringetin-dihexoside), three flavan-3-ols (−)-epicatechin, (+)-catechin, (−)-epicatechin gallate) and four proantocyanidins (procyanidin B1, procyanidin B2, procyanidin B3 or B4/B5, procyanidin T2 or T3/T4/C1). Seibel 19881, Seyve Villard 12-347 and Seyve Villard 29-399 were particularly rich in polyphenols. These findings emphasize that these grapes are especially interesting for the production of antioxidant extracts for nutraceutical and pharmaceutical uses.

## 1. Introduction

The health benefits of grape and wine consumption are mainly associated with their polyphenolic content (the so-called *French paradox* [1]). Polyphenols are antioxidant compounds that act as reducing agents by donating hydrogen, acting as chelators, quenching singlet oxygen and by trapping free radicals [2]. Moreover, their activity in reducing coronary heart diseases, atherosclerosis [3,4], various types of cancer, and dermal disorders, was demonstrated [5,6].

Grape polyphenols can be divided in anthocyanic and non-anthocyanic compounds. The latter mainly include flavan-3-ols and their oligomers (proanthocyanidins and tannins), glycoside flavonols, hydroxycinnamates, hydroxybenzoates, and stilbenes derivatives [7].

Main flavan-3-ols in grape are catechin, epicatechin, gallocatechin, epigallocatechin, and epicatechin-3-*O*-gallate [7,8,9]. These compounds are located in the berry skin and seeds and are potent antioxidants characterized by important biological, pharmacological and medicinal properties: e.g., they are proven to protect human low density lipoprotein (LDL) against oxidation more efficiently than α-tocopherol on a molar basis, and act as cardioprotective agents [6,10,11,12].

Flavonols are located in the skin where are involved in the shielding from UV rays [13]. Principal are myricetin, quercetin and kaempferol mainly present as glucoside and glucuronide derivatives [14]. As well as glucoside, laricitrin, isorhamnetin and syringetin were found in grape in galactoside and glucuronide forms [15,16,17]. Flavonols are antioxidant and bioactive compounds present in dietary plants used for human nutrition [18]. For example, *in vitro* studies showed quercetin inhibits human platelet aggregation and acts as potential anticancer by inhibiting carcinogens and cancer cell growth in many experimental and human tumors [19,20].

Tannins are divided in condensed and hydrolysable. Condensed tannins (proanthocyanidins) are flavan-3-ol polymers mainly present in the solid parts of grape (skin and seeds) and, in minor amount, in the pulp [21]. *In-vivo* studies showed that grape seed procyanidin extract has an antioxidant activity as important as vitamin E, by preventing oxidative damage of tissues by reduction of the lipid oxidation and/or inhibition of free radicals production [22].

Traditional analytical methods for analysis of low molecular weight (MW) flavonoids are performed by reverse-phase high performance liquid chromatography (HPLC) coupled with UV-Vis spectrophotometry or mass spectrometry (MS) [14,23]. In general, liquid chromatography/mass spectrometry (LC/MS) and multiple mass spectrometry (MS/MS and MS^n^) are effective for the structural characterization [24]. LC coupled to high-resolution mass spectrometry (HRMS) has proven to be very effective in grape metabolomics, in particular for characterization of stilbene derivatives, flavonols and anthocyanins [25]. Recently, a database for grape and wine metabolomics (*GrapeMetabolomics*), which currently contains around 1100 putative compounds identified by HRMS, was constructed [26].

In previous studies, some hybrid grape varieties were highlighted as particularly interesting for their qualitative and quantitative anthocyanin and flavonol profiles [27,28]. In the present work, the study of more promising varieties was extended to the other non-anthocyanic flavonoids to complete their evaluation as sources of bioactive and antioxidant compounds potentially useful for industrial purposes. Polyphenols of their seed extracts were recently investigated [29], as a consequence the study was focalized on the skin/pulp extract. Characterization of compounds by ultra-high performance-liquid chromatography/quadrupole-time of flight mass spectrometry (UHPLC/QTOF) analysis, was performed.

## 2. Results and Discussion

**Figure 1:**
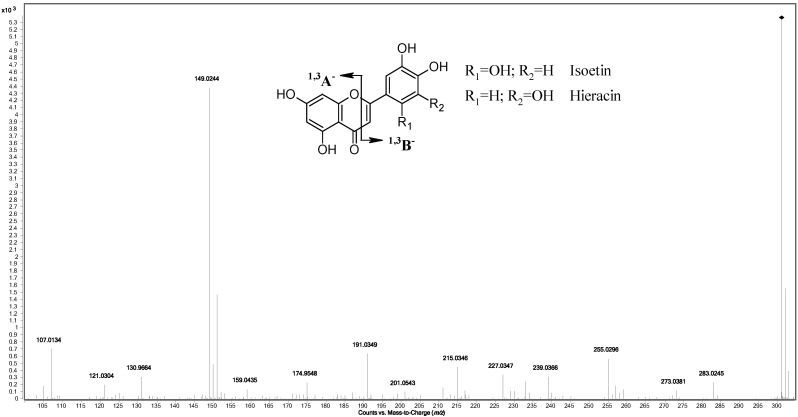
MS/MS spectrum of putative quercetin isomer flavone-type identified.

In high-resolution MS metabolomics, the identification of the molecular formula (MF) of compounds relies on the measurement of the mono-isotopic mass and isotopic pattern (relative abundances and *m*/*z* distances) followed by the raw data processing performed by using specific algorithms [30,31]. In this study, the first “targeted” identification of compounds was performed by comparing the theoretical isotopic pattern of the metabolites present in *GrapeMetabolomics* with the experimental ones calculated by the algorithms using the signals measured. Then, “untargeted” data processing of the remaining raw data was performed and provided the identification of possible other compounds not included in the database. Identification of compounds was based on the [M − H]^−^ ion isotopic pattern and confirmed by MS/MS analysis. In the five grape samples, thirty-one non-anthocyanic flavonoids reported in Table 1 were identified including nineteen flavonols, two flavanonols, three flavan-3-ols, two flavanones, one flavone and four proanthocyanidins. Among them, methylnaringenin, pentahydroxy flavone quercetin isomer, syringetin-dihexoside, and tetrahydroxy-dimethoxyflavanone-hexoside were identified by the “untargeted” method. Almost all the compounds were identified with low mass error (maximum 2.7 ppm) and identification scores >90%. Just three of them—dihydrokaempferol-3-*O*-rhamnoside, kaempferide-*p*-coumaroylhexoside, and isorhamnetin-glucuronide—had a lower identification score (73%, 80% and 79%, respectively) probably due to their low signal intensity. For glucoside and glucuronide flavonols different characteristic fragmentations, were observed: glucoside compounds showed high signal of radical aglycone ion [Y_0_ − H]^−•^ instead the glucuronide derivatives had higher signal of aglycone ion Y_0_^−^. A putative quercetin isomer was also identified: main MS/MS fragments at *m*/*z* 149.0244, which corresponds to the formula C_8_H_5_O_3_, and at *m*/*z* 151.0035 of ion C_7_H_3_O_4_, were observed (Table 1). Potentially, they form by ^1,3^B^−^ fragmentation of flavones and flavonols [32] and the putative structure proposed for the compound is shown in Figure 1.

By performing analysis of samples collected in two consecutive vintages (harvests 2011 and 2012) it was possible to estimate the semi-quantitative content of the compounds reported in Table 2. Among the compounds identified two new flavonoids—isorhamnetin-*p*-coumaroylglucoside and kaempferide-*p*-coumaroylhexoside—were recently found in grape [33]. They were more abundant in the samples Siebel 19881 and Siebel 8357 (Table 2).

**Table 1:** [M − H]^−^ ion of non-anthocyanic flavonoids identified in the five hybrid grape samples studied. ppm: mass error; Id score: overall identification score calculated on the isotopic pattern signals of the compound. MS/MS, main identification fragments (underlined, the more abundant).

Flavonoids Identified	Rt	Formula	[M − H]^−^ Ion		Error	Id Score	MS/MS
	(min)		Experimental Theoretical		(ppm)	(%)	*m*/*z*
(−)-epicatechin	13.63	C_15_H_14_O_6_	289.0723	289.0718	1.7	97.8	205.0502 151.0399
(+)-catechin	12.61	C_15_H_14_O_6_	289.0715	289.0718	−1.0	99.3	205.0500 151.0396
(−)-epicatechin-3-*O*-gallate	15.11	C_22_H_18_O_10_	441.0831	441.0827	0.9	98.3	289.0719 169.0142
dihydrokaempferol-3-*O*-rhamnoside	15.98	C_21_H_22_O_10_	433.1148	433.1140	1.8	73.6	287.0563 269.0457
dihydroquercetin-3-*O*-hexoside	14.34	C_21_H_22_O_12_	465.1048	465.1038	2.1	90.0	303.0512 151.0040
isorhamnetin-3-*O*-glucoside	15.72	C_22_H_22_O_12_	477.1045	477.1038	1.5	98.5	314.0431 271.0242
isorhamnetin-glucuronide	15.90	C_22_H_20_O_13_	491.0842	491.0831	2.2	79.7	315.0514 271.0243
isorhamnetin-*p*-coumaroylglucoside	17.67	C_31_H_28_O_14_	623.1412	623.1406	0.9	93.8	477.1021 315.0511
kaempferide-*p*-coumaroylhexoside	17.80	C_31_H_28_O_13_	607.1459	607.1457	0.3	80.4	461.1088 299.0557
kaempferol-3-*O*-galactoside	15.41	C_21_H_20_O_11_	447.0942	447.0933	2.0	93.2	284.0337 255.0305
kaempferol-3-*O*-glucoside	15.61	C_21_H_20_O_11_	447.0943	447.0933	2.2	94.3	284.0333 255.0299
laricitrin-3-*O*-glucoside	15.01	C_22_H_22_O_13_	493.0994	493.0988	1.2	98.0	330.0377 315.0143
methylnaringenin	22.37	C_16_H_14_O_5_	285.0773	285.0768	1.7	98.3	270.0539 164.0116
myricetin-3-*O*-glucoside	14.27	C_21_H_20_O_13_	479.0845	479.0831	2.9	95.3	316.0233 271.0248
myricetin-3-*O-*glucuronide	14.20	C_21_H_18_O_14_	493.0622	493.0624	−0.4	98.9	317.0303 271.0242
myricetin-dihexoside	14.11	C_27_H_30_O_18_	641.1362	641.1359	0.5	99.5	479.0838 316.0231
myricetin-glucoside-glucuronide	14.10	C_27_H_28_O_19_	655.1153	655.1152	0.1	95.0	479.0815 317.0299
pentahydroxy flavone (isom. quercetin)	16.66	C_15_H_10_O_7_	301.0356	301.0354	0.7	99.7	149.0244 151.0035
procyanidin B1	12.02	C_30_H_26_O_12_	577.1348	577.1351	−0.5	99.2	407.0772 289.0718
procyanidin B3/B4/B5	12.35	C_30_H_26_O_12_	577.1345	577.1351	−1.0	94.6	407.0779 289.0724
procyanidin B2	13.25	C_30_H_26_O_12_	577.1353	577.1351	0.3	95.1	407.0771 289.0716
procyanidin T2/T3/T4/C1	12.84	C_45_H_38_O_18_	865.1987	865.1985	0.2	91.4	577.1349 289.0716
quercetin	18.01	C_15_H_10_O_7_	301.0356	301.0354	0.7	99.5	273.0406 151.0040
quercetin-3-*O*-galactoside	14.91	C_21_H_20_O_12_	463.0892	463.0882	2.2	97.1	300.0284 151.0038
quercetin-3-*O*-glucoside	15.02	C_21_H_20_O_12_	463.0889	463.0882	1.5	98.4	300.0284 151.0036
quercetin-3-*O*-glucuronide	14.96	C_21_H_18_O_13_	477.0688	477.0675	2.7	96.5	301.0360 151.0036
rutin (querc-3-*O*-rutinoside)	14.63	C_27_H_30_O_16_	609.1467	609.1461	1.0	97.4	463.0877 300.0279
syringetin-3-*O*-galactoside	15.53	C_23_H_24_O_13_	507.1147	507.1144	0.6	94.7	345.0623 330.0390
syringetin-3-*O*-glucoside	15.68	C_23_H_24_O_13_	507.1148	507.1144	0.8	98.1	344.0536 329.0298
syringetin-dihexoside	13.94	C_29_H_34_O_18_	669.1672	669.1672	0.0	99.7	507.1144 345.0621
tetrahydroxy-dimethoxyflavanone-hexoside	14.19	C_23_H_26_O_13_	509.1308	509.1301	1.4	98.6	346.0694 329.0674

**Table 2:** Semi-quantitative contents of non-anthocyanic flavonoids identified in the samples. Mean data of four samples (harvests 2011 and 2012), are reported.

Flavonoids	Unknown Red	Seibel 19881	Seyve Villard 12-347	Seyve Villard 29-399	Seibel 8357
			μg/kg Grape		
**Flavan-3-ols**					
(−)-epicatechin ^a^	4555 ± 1631	6117 ± 241	7467 ± 3491	11640 ± 2133	7126 ± 2447
(+)-catechin ^b^	5578 ± 1173	13186 ± 802	11450 ± 3299	13257 ± 813	1032 ± 531
(−)-epicatechin-3-*O*-gallate ^c^	1140 ± 146	1028 ± 293	673 ± 154	2111 ± 270	674 ± 74
**Total flavan-3-ols**	**11273**	**20331**	**19590**	**27009**	**8832**
**Flavanonols**					
dihydrokaempferol-3-*O*-rhamnoside ^d^	15 ± 1	1082 ± 155	25 ± 4	374 ± 88	19 ± 2
dihydroquercetin-3-*O*-hexoside ^e^	40 ± 7	2602 ± 680	n.d.	224 ± 94	n.d.
**Flavonols**					
isorhamnetin-3-*O*-glucoside ^f^	46 ± 15	1090 ± 119	591 ± 192	258 ± 26	254 ± 56
isorhamnetin-glucuronide ^f^	n.d.	6 ± 1	n.d.	n.d.	n.d.
isorhamnetin-*p*-coumaroylglucoside ^f^	n.d.	46 ± 4	7 ± 2	n.d.	22 ± 5
kaempferide-*p*-coumaroylhexoside ^d^	n.d.	39 ± 8	n.d.	n.d.	34 ± 1
kaempferol-3-*O*-galactoside ^d^	9 ± 0	579 ± 10	933 ± 51	45 ± 9	36 ± 15
kaempferol-3-*O*-glucoside ^d^	29 ± 5	2124 ± 75	2456 ± 47	161 ± 40	162 ± 78
laricitrin-3-*O*-glucoside ^e^	560 ± 5	1681 ± 165	410 ± 60	177 ± 50	3240 ± 1240
myricetin-3-*O*-glucoside ^h^	26702 ± 511	41569 ± 2324	24859 ± 4236	12458 ± 2148	35751 ± 1204
myricetin-3-*O*-glucuronide ^h^	269 ± 32	202 ± 12	68 ± 1	50 ± 2	154 ± 23
myricetin-diglucoside ^h^	538 ± 137	376 ± 74	612 ± 103	334 ± 70	682 ± 5
myricetin-glucoside-glucuronide ^h^	46 ± 8	23 ± 2	n.d.	n.d.	n.d.
pentahydroxy flavone (isom. quercetin) ^f^	98 ± 22	444 ± 125	100 ± 23	75 ± 46	522 ± 50
quercetin ^f^	34 ± 8	316 ± 29	182 ± 49	52 ± 11	119 ± 45
quercetin-3-*O*-galactoside ^e^	159 ± 2	5124 ± 331	4803 ± 452	705 ± 338	1027 ± 637
quercetin-3-*O*-glucoside ^e^	2347 ± 380	12559 ± 69	16381 ± 548	6943 ± 1928	6124 ± 1758
quercetin-3-*O*-glucuronide ^e^	618 ± 21	11411 ± 33	2501 ± 610	1142 ± 395	2220 ± 896
rutin (querc-3-*O*-rutinoside) ^h^	n.d	13468 ± 1381	9887 ± 1496	2747 ± 1337	6091 ± 3070
syringetin-3-*O*-galactoside ^f^	13 ± 0	57 ± 15	21 ± 2	22 ± 8	25 ± 4
syringetin-3-*O*-glucoside ^f^	252 ± 70	1823 ± 139	228 ± 29	350 ± 49	1327 ± 388
syringetin-dihexoside ^f^	8 ± 2	176 ± 49	37 ± 6	39 ± 18	18 ± 7
Total flavonols *	**31632**	**92669**	**63976**	**25484**	**57285**
**Procyanidins**					
procyanidin B1 ^i^	3638 ± 903	2698 ± 515	7418 ± 1253	9698 ± 2364	1528 ± 636
procyanidin B3/B4/B5 ^i^	1117 ± 127	1332 ± 81	3929 ± 1363	3913 ± 110	2684 ± 886
procyanidin B2 ^l^	3836 ± 445	1603 ± 212	5029 ± 2393	6135 ± 4	634 ± 129
procyanidin T2/T3/T4/C1 ^i^	339 ± 17	389 ± 32	617 ± 246	849 ± 17	591 ± 166
**Total procyanidins**	**8930**	**6022**	**16992**	**20596**	**5437**
**Flavanones**					
methylnaringenin ^g^	114 ± 7	203 ± 7	88 ± 13	114 ± 11	136 ± 2
tetrahydroxy-dimethoxyflavanone-hexoside ^f^	949 ± 294	3600 ± 630	910 ± 546	530 ± 398	7427 ± 449
**Total flavonoids**	**53052**	**126953**	**101681**	**74406**	**79660**

Superindexes indicate the standard used to calculate the concentration: ^a^ concentration calculated with analytical response factor of (−)-epicatechin; ^b^ (+)-catechin; ^c^ (−)-epicatechin gallate; ^d^ kaempferol-3-*O*-glucoside; ^e^ quercetin-3-*O*-glucoside; ^f^ isorhamnetin-3-*O*-glucoside; ^g^ 4ʹ,5,7-trihydroxy flavanone; ^h^ myricetin glucoside; ^i^ procyanidin B1; ^l^ procyanidin B2. *, pentahydroxy flavone (isom. quercetin) was not counted.

Currently, the grape varieties studied are not used for production and just five plants of each variety are present in the CREA-VIT vine collection. As a consequence, a quantitative study really representative of the compounds produced in the grape was not possible. Anyway, a semi-quantitative analysis of the compounds was performed in order to have main information on their presence in the grape. For some compounds, such as catechin, rutin, some quercetin derivatives, high variability was observed (±50%, Table 2), mainly due to the differences between two vintages. In general, flavonols were the most abundant non-anthocyanic flavonoids identified with a total content between 25 and 93 mg/kg calculated as sum of the single compounds in Table 2 (34%–73% of the total non-anthocyanic flavonoids identified). Total flavan-3-ol content was between 9 and 27 mg/kg (11%–36% of the total non-anthocyanic flavonoids) and proanthocyanidins between 5 and 20 mg/kg (5%–27% of the non-anthocyanic flavonoids). In a previous study, high contents of flavonols were found also in other hybrid red grape varieties, such as Seibel 9280 and Seibel 6339, and the white grape Seyve-Villard 12.303 [34]. In all samples, the flavonol profile was dominated by myricetin (myricetin-3-*O*-glucoside between 12.4 and 41.5 mg/kg) and the highest flavanol was procyanidin B1 (between 1.5 and 9.7 mg/kg). Similarly, high contents of procyanidin B1 and myricetin-3-*O*-glucoside were found in the *V. labrusca* grape variety Bordô and in skin of the table grape varieties BRS Clara and BRS Morena [35,36]. Myricetin-3-*O*-glucoside was the more abundant flavonol also in the hybrid grape cultivar BRS Violeta, but in this case the main flavanol was (+)-catechin in both skin and flesh [37].

Among the samples studied, Seibel 19881 had the highest total content of flavonols (93 mg/kg) and of flavanonols (3.7 mg/kg). In particular, this variety had considerable amounts of quercetin (43 mg/kg), myricetin (42 mg/kg), syringetin (2.0 mg/kg), and isorhamnetin (1.1 mg/kg). Moreover, this sample was characterized by relevant content of isorhamnetin-*p*-coumaroylglucoside (46 μg/kg), a compound recently identified in Raboso Piave *V. vinifera* grape together with other two *p*-coumaroylglucoside flavonoids (dihydrokaempferide-3-*O*-*p*-coumaroylhexoside and kaempferide-*p*-coumaroylhexoside) [33], by the presence of isorhamnetin-glucuronide, and significant contents of dihydrokaempferol-3-*O*-rhamnoside, dihydroquercetin-3-*O*-hexoside, and myricetin-3-*O*-glucoside-glucuronide. Both Seibel grape samples had high contents of pentahydroxy flavone and tetrahydroxy-dimethoxyflavanone-hexoside. Seyve Villard 29-399 was characterized by the highest contents of total flavan-3-ols and proantocyanidins, in particular (−)-epicatechin (11.6 mg/kg), (+)-catechin (13.2 mg/kg), epicatechin-3-*O*-gallate (2.1 mg/kg), procyanidin B1 and procyanidin B2 (9.7 mg/kg and 6.1 mg/kg, respectively) (Table 2). Also the other Seyve Villard sample (Seyve Villard 12-347) was characterized by high procyanidin B1 and procyanidin B2 (7.4 mg/kg and 5.0 mg/kg, respectively), (−)-epicatechin and (+)-catechin (7.5 mg/kg and 11.4 mg/kg, respectively), and by the highest contents of quercetin-3-*O*-glucoside (16.4 mg/kg) and kaempferol-3-*O*-glucoside (2.4 mg/kg).

## 3. Experimental Section

### 3.1. Chemicals, Samples and Sample Preparation

Standards of myricetin-3-*O*-glucoside, quercetin, quercetin-3-*O*-glucoside, isorhamnetin-3-*O*-glucoside, kaempferol-3-*O*-glucoside, rutin, procyanidin B1, procyanidin B2, (+)-catechin, (−)-epicatechin, (−)-epicatechin-3-*O*-gallate, (−)-epigallocatechin gallate, (−)-epigallocatechin were purchased from Extrasynthese (Genay, France); 4ʹ,5,7-trihydroxyflavanone was purchased from Sigma-Aldrich (Milan, Italy). Grape samples studied were: An “unknown hybrid red grape variety”, Seibel 8357 (Seibel 6150 × Seibel 5455: Seibel 6150 = {[(*V. lincecumii* × *V. rupestris*) × *V. vinifera*] × (Aramon × Ganzin)} × [(*V. lincecumii* × *V. rupestris*) × Aramon]; Seibel 5455 = {[(Aramon × Ganzin) × Bouboulenc (*V. vinifera*)] × [(*V. lincecumii* × *V. rupestris*) × (*V. aestivalis* × *V. cinerea*)] × *V. berlandieri*}; *V*IVC number 2768, http://www.vivc.de/), Seibel 19881 (unknown; *V*IVC number 11461), Seyve Villard 29-399 (unknown; *V*IVC number 11663) and Seyve Villard 12-347 (Seibel 6468 × Seibel 6905: Seibel 6468 = {[(Aramon × Ganzin) × Bouboulenc] × Sicilien (*V. vinifera*)]} × {[(*V. lincecumii* × *V. rupestris*) × (*V. aestivalis* × *V. cinerea*)] *× V. berlandieri*} × [(*V. vinifera* × *V. labrusca*) × *V. rupestris*] × *V. vinifera*; Seibel 6905 = Seibel 451 (unknown; *V*IVC number 10942) × {[(*V. lincecumii* × *V. rupestris*) × *V. vinifera*] × (Aramon × Ganzin)} × {[(*V. lincecumii* × *V. rupestris*) × *V. vinifera*] × (Aramon × Ganzin)} × {[(*V. rupestris*) × *V. rupestris*)] × Blanc Royal}; *V*IVC number 11587). Grapes were harvested in 2011 and 2012 at ripeness from the five plants of each variety present in the CREA-VIT grapevine germplasm repository (Susegana, Veneto, Italy). Mean sugar content of samples at the harvests were: Unknown hybrid red grape variety °Brix = 18.2 ± 0.8; Seibel 8357 °Brix= 19.4 ± 1.0; Seibel 19881 °Brix = 21.3 ± 1.1; Seyve Villard 29-399 °Brix = 17.7 ± 0.2; Seyve Villard 12-347 °Brix = 18.9 ± 1.0. For each sample, about 100 berries were picked randomly and immediately frozen at −20 °C. For sample preparation, twenty berries were weighed and homogenized using liquid nitrogen and the resulting powder was immediately extracted with pure methanol in ratio 2:1 *v*/*w* under stirring for 20 min in the dark. After addition to the extract of 200 μg/L of a 4ʹ,5,7-trihydroxyflavanone 500 mg/L solution as internal standard, the vial sample was wrapped with an aluminum foil to limit possible photo-oxidation and centrifuged at 10 °C for 20 min. The solution was filtered with an Acrodisc GHP 0.22 μm filter (Waters, Milford, MA, USA) and collected in a vial for LC/MS analysis. To perform quantitative analysis a standard solution was analyzed and RF_IS_/RF_analyte_ ratio of each compound was calculated. RF_IS_/RF_analyte_ coefficients were: Procyanidin B1 = 29.2, procyanidin B2 = 58.4; quercetin = 2.4; quercetin-3-*O*-glucoside = 8.2; rutin = 12.7; isorhamnetin-3-*O*-glucoside = 2.4; kaempferol-3-*O*-glucoside = 2.5; myricetin-3-*O*-glucoside = 14.1; (+)-catechin = 5.8; (−)-epicatechin = 6.6; (−)-epicatechin gallate = 16.7; (−)-epigallocatechin gallate = 64.3; (−)-epigallocatechin = 11.1.

### 3.2. LC/QTOF Mass Spectrometry

The analytical system used was Agilent UHPLC 1290 Infinity coupled to Agilent 1290 Infinity Autosampler (G4226A) and Agilent 6540 accurate-mass Q-TOF mass spectrometer (nominal resolution 40.000) with Jet Stream Ionization source (Agilent Technologies, Santa Clara, CA, USA). For each sample, two negative ionization mode analyses were performed with full scan acquisition. After each sample a blank was run. The data acquisition software was Agilent MassHunter version B.04.00 (B4033.2). Chromatography was performed using a Zorbax reverse-phase column (RRHD SB-C18 3 mm × 150 mm, 1.8 μm) (Agilent Technologies). The mobile phase was composed of (A) 0.1% *v*/*v* aqueous formic acid; and (B) 0.1% *v*/*v* formic acid in acetonitrile. Gradient elution program: 5% B isocratic for 8 min, from 5% to 45% B in 10 min, from 45% to 65% B in 5 min, from 65% to 90% in 4 min, and 90% B isocratic for 10 min. Flow rate was 0.4 mL/min; sample injection was 10 μL; column temperature was 35 °C.

QTOF conditions: sheath gas nitrogen 10 L/min at 400 °C, drying gas 8 L/min at 350 °C, nebulizer pressure 60 psi, nozzle voltage 1 kV, and capillary voltage 3.5 kV. Signals in the *m/z* 100–1700 range were recorded. Negative mass calibration was performed with standard mix G1969-85000 (Supelco Inc., Bellefonte, PA, USA) and had residual error for the expected masses between ±0.2 ppm. Lock masses were: TFA anion at *m*/*z* 112.9856 and HP-0921(+formate) at *m*/*z* 966.0007 in negative-ion mode.

MS/MS conditions: collision energies between 20 and 60 eV were used to fragment ions in the *m*/*z* 100–1700 range. Acquisition rate was 2 spectra/s.

Data processing was performed with Agilent Mass Hunter Qualitative Analysis software version B.05.00 (5.0.519.0) (Agilent Technologies). Confidence of the compound identification was based on accurate mass and isotope pattern and was expressed by an “overall identification score” computed as a weighted average of the compound isotopic pattern signals, such as exact masses, relative abundances, and *m*/*z* distances (spacing). Weight parameters were W_mass_ = 100, W_abundance_ = 60, and W_spacing_ = 50; mass expected data variation 2.0 mDa + 5.6 ppm, mass isotope abundance 7.5%, and mass isotope grouping peak spacing tolerance 0.0025 *m*/*z* + 7.0 ppm.

## 4. Conclusions

Among the samples studied, Seibel 19881 and the two Seyve Villard varieties are characterized by peculiar richness of non-anthocyanic flavonoids. In particular, Seyve Villard samples have high content of flavan-3-ols, procyanidin B1 and procyanidin B2. It is worth to highlight that these compounds have protective activity towards LDL oxidation greater than standard antioxidants like trolox and ascorbic acid [38]. Seibel 19881 is rich in antioxidant compounds, such as flavonols and flavanonols, and has relevant content of high isorhamnetin derivatives.

A previous study showed that these Seyve Villard grape varieties have low anthocyanin content [27]. On the other hand, these findings emphasize that they can be interesting for extraction of non-anthocyanic antioxidant compounds. Seibel 19881 confirmed its richness of non-anthocyanic polyphenols by showing that it is particularly interesting for production of extracts for nutraceutical and pharmaceutical uses. Despite in the previous study grape Seibel 8357 showed the highest anthocyanins content, this variety is not particularly rich in non-anthocyanic flavonoids, even if is characterized by relevant syringetin content. This step of the research was to investigate the presence in grape of non-anthocyanic flavonoids potentially interesting for nutraceutical purposes. If an industrial interest should arise, separated analysis of skins and pulps will have to be performed.
